# Capsule Promotes Intracellular Survival and Vascular Endothelial Cell Translocation during Invasive Pneumococcal Disease

**DOI:** 10.1128/mBio.02516-21

**Published:** 2021-10-12

**Authors:** Terry Brissac, Eriel Martínez, Katherine L. Kruckow, Ashleigh N. Riegler, Feroze Ganaie, Hansol Im, Sayan Bakshi, Nicole M. Arroyo-Diaz, Brady L. Spencer, Jamil S. Saad, Moon H. Nahm, Carlos J. Orihuela

**Affiliations:** a Department of Microbiology, School of Medicine, University of Alabama at Birmingham, Birmingham, Alabama, USA; b Division of Pulmonary, Allergy, and Critical Care Medicine, Department of Medicine, University of Alabama at Birmingham, Birmingham, Alabama, USA; University of Illinois at Chicago

**Keywords:** *Streptococcus pneumoniae*, pathogenesis, virulence, invasive disease, capsular polysaccharide, capsule, endothelial cells, antioxidant, reactive oxygen species, intracellular bacteria

## Abstract

The polysaccharide capsule that surrounds Streptococcus pneumoniae (*Spn*) is one of its most important virulence determinants, serving to protect against phagocytosis. To date, 100 biochemical and antigenically distinct capsule types, i.e., serotypes, of *Spn* have been identified. Yet how capsule influences pneumococcal translocation across vascular endothelial cells (VEC), a key step in the progression of invasive disease, was unknown. Here, we show that despite capsule being inhibitory of *Spn* uptake by VEC, capsule enhances the escape rate of internalized pneumococci and thereby promotes translocation. Upon investigation, we determined that capsule protected *Spn* against intracellular killing by VEC and H_2_O_2_-mediated killing *in vitro*. Using a nitroblue tetrazolium reduction assay and nuclear magnetic resonance (NMR) analyses, purified capsule was confirmed as having antioxidant properties which varied according to serotype. Using an 11-member panel of isogenic capsule-switch mutants, we determined that serotype affected levels of *Spn* resistance to H_2_O_2_-mediated killing *in vitro*, with killing resistance correlated positively with survival duration within VEC, rate of transcytosis to the basolateral surface, and human attack rates. Experiments with mice supported our *in vitro* findings, with *Spn* producing oxidative-stress-resistant type 4 capsule being more organ-invasive than that producing oxidative-stress-sensitive type 2 capsule during bacteremia. Capsule-mediated protection against intracellular killing was also observed for Streptococcus pyogenes and Staphylococcus aureus. We conclude that capsular polysaccharide plays an important role within VEC, serving as an intracellular antioxidant, and that serotype-dependent differences in antioxidant capabilities impact the efficiency of VEC translocation and a serotype’s potential for invasive disease.

## INTRODUCTION

Streptococcus pneumoniae (*Spn*) is the leading cause of community-acquired pneumonia. As result, it is also a leading cause of invasive infections, including bacteremia and meningitis. Young infants, the elderly, immunocompromised individuals, and those who are experiencing or have recently overcome a viral respiratory tract infection, are particularly susceptible to the severe pneumonia that can lead to invasive pneumococcal disease (IPD) (1, 2). Critically, mortality rates for the elderly with hospital-admitted pneumococcal pneumonia with bacteremia can be as high as 60% (3, 4). Thus, IPD is a major medical problem. It is noteworthy that not all *Spn* are equally capable of causing IPD. To date, 100 biochemical and antigenically distinct capsule types, i.e., serotypes, of *Spn* have been identified (5), of which only 25 to 30 are commonly associated with human disease (6, 7). Thus, the most obvious determinant of *Spn* disease propensity is the serotype of the infecting strain.

Polysaccharide capsule is a primary virulence determinant of *Spn* and numerous other pathogens (8). Nonencapsulated *Spn* can cause disease, but this is almost never life-threatening and is generally restricted to the upper respiratory tract or the eye (9, 10). The reason for this is that capsule protects the bacterium from host clearance via inhibition of complement deposition and by obscuring bacterial surface-attached host defense factors from their cognate receptors on immune cells (e.g., Fc portion of antibody), thereby blocking opsonophagocytosis (11, 12). In addition, the vast majority of *Spn* capsule types are negatively charged and electrostatically repel host cells, whose surfaces are also negatively charged due to the presence of anionic glycoconjugates (13–15). Importantly, and for many of the same reasons, capsule starkly impairs bacterial adhesion and invasion of nonimmune cells, an interaction that is critical for *Spn* colonization, the development of pneumonia, and progression to disseminated infection (15–18). Thus, *Spn* must have means to counter the inhibitory effect of its own capsule when appropriate. The ways that *Spn* does this include phase variation, which alters the amount of capsule produced (19), autolysin-mediated capsule shedding when exposed to LL-37, the result of proximity to mucosal epithelial cells (18, 20), and the incorporation of stalk-like elements within surface proteins to allow for the extension of adhesion domains beyond the capsule layer (21). Importantly, and in contrast to the airway, pneumococci within the bloodstream must be heavily encapsulated, as without capsule, they are rapidly opsonized by serum factors and cleared by phagocytes (22). Thus, capsule is present when *Spn* in the bloodstream interact with vascular endothelial cells (VEC) at the blood brain barrier or in capillaries of other organs (23).

*Spn* is the prototypical extracellular pathogen. Yet we also know that *Spn* can be taken up by VEC. Uptake of *Spn* by VEC has been demonstrated to occur via platelet-activating factor receptor-initiated clathrin-mediated endocytosis (CME) and results in either bacterial degradation within the endolysosome, return to the apical surface, or translocation of bacteria across the cell and their release at the basolateral surface (24–26). Whereas CME-instigated translocation across VEC at the blood-brain barrier has long been considered to be a pivotal step in the pathogenesis of bacterial meningitis (25), this process is now also appreciated as having an instigating role in other aspects of disseminated organ damage during bacteremia, such as myocardial invasion and cardiac damage (27). Pneumococcal adhesins involved in the VEC translocation process include cell wall phosphorylcholine, which binds to platelet-activating factor receptor (24), choline binding protein A, which binds host laminin receptor and polymeric immunoglobulin receptor (pIgR) (28, 29), and the pneumococcal pilus, which binds pIgR as well as PECAM-1 (29). It is noteworthy that since capsule has such a pronounced inhibitory effect on these interactions (25), the bulk of work done *in vitro* to characterize *Spn* translocation has been done using unencapsulated mutants.

Here, and in stark contrast with the notion that the capsule is inhibitory of *Spn* invasion processes, we demonstrate that encapsulated pneumococci are more efficient at translocation across VEC once they have been internalized. What is more, we identify a new role for this classic virulence determinant, as an intracellular antioxidant. We show that capsule prolongs *Spn* survival within VEC and enhances the rate of translocation across cell barriers *in vitro* and into organs *in vivo* and that the extent to which different serotypes confer an antioxidant effect correlates with their propensity for invasive disease in humans. We also extend our findings to other encapsulated bacterial pathogens and thereby advance our overall understanding of bacterial pathogenesis and the molecular basis for disseminated disease.

## RESULTS

### Capsule mediates *Spn* transmigration through a vascular endothelial cell layer.

**FIG 1:**
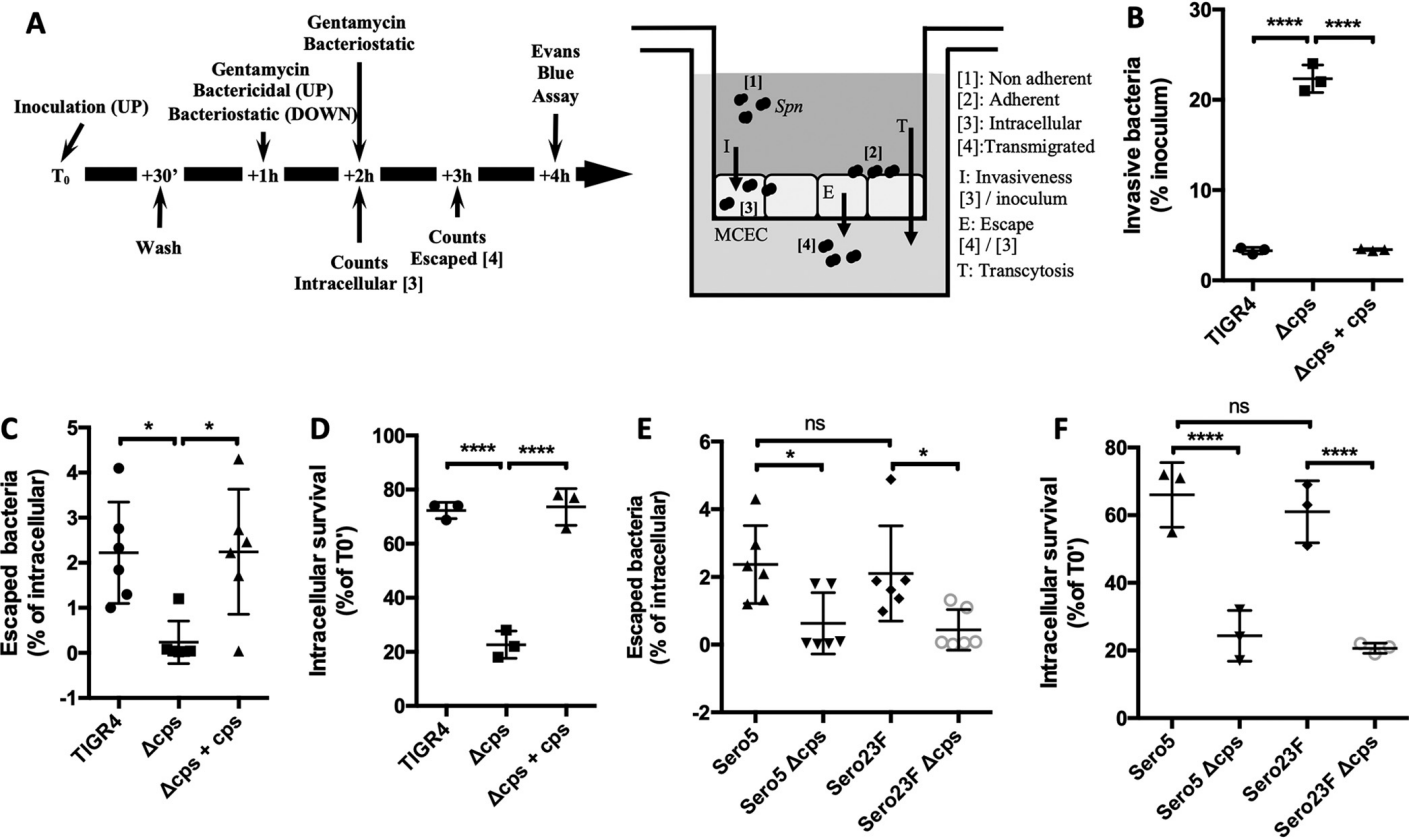
Capsule increases intracellular survival and bacterial escape from vascular endothelial cells. (A) Experimental flow chart and Transwell system setup with MCEC. (B) Encapsulated WT *Spn* (TIGR4) and a complemented mutant (Δ*cps* + *cps*) were internalized at lower rates but, once within cells (C), escaped with greater frequency than the isogenic nonencapsulated mutant (Δ*cps*). (D) Capsule promotes *Spn* intracellular survival. Intracellular survival of designated bacterial strains was determined by normalizing CFU at *T*_0_ + 2 h of treatment by CFU at *T*_0_ + 1 h of treatment (*T*_0_′). Clinical isolates corresponding to serotypes 5 and 23F were tested alongside their unencapsulated derivatives for rates of (E) MCEC escape and (F) intracellular survival. Statistical analyses: Mann-Whitney U-test (panels B to F). Each dot is a biological replicate. Errors bars represent the standard error of the mean. Asterisks denote statistical significance: ns, not significant; *, *P < *0.05; ****, *P < *0.0001.

Since *Spn* in the bloodstream is encapsulated when they come in contact with VEC, and VEC translocation is a key event in the development of disseminated infection, we sought to determine how capsule impacts the bacterium’s fate following internalization by VEC. To do so, we developed an *in vitro* Transwell assay using mouse cardiovascular endothelial cells (MCEC) that allowed for stepwise analyses of the bacterial transmigration process (Fig. 1A). MCEC were chosen as the prototype host cell since (i) they formed confluent leak-free monolayers (Fig. S1), (ii) during bacteremia *Spn* must cross this cell type to invade the myocardium to cause cardiac damage, and (iii) bacterial translocation across MCEC is platelet-activating factor receptor and laminin receptor dependent (27), consistent with CME as the means for bacterial translocation (26). Similar to prior published work (16, 18, 30), we observed that an isogenic capsule deletion mutant of serotype 4 strain TIGR4 (Δ*cps*) was internalized by MCEC at a greater rate, >5-fold, than wild-type (WT) TIGR4 or the *cps* complemented strain (Δ*cps* + *cps*) (Fig. 1B). Yet unexpectedly, and despite this lower invasion rate, the percentage of internalized WT TIGR4 or Δ*cps* + *cps* determined to successfully passage to the basolateral surface of the monolayer was >10-fold greater than Δ*cps* (Fig. 1C). We postulated that capsule enhanced VEC translocation by prolonging bacterial survival. In support of this notion, gentamicin protection assays which specifically kill extracellular bacteria (31) showed that after 2 h of treatment the number of viable *Spn* recoverable from within MCEC was >3-fold higher for WT TIGR4 versus Δ*cps* (Fig. 1D). Similar experiments were performed using low-passage-number clinical isolates belonging to serotypes 5 and 23F and their respective unencapsulated mutants (Fig. 1E and F). The observation that capsule belonging to these serotypes also promoted escape of pneumococci from within VEC and increased the percentage of internalized *Spn* that remained alive suggested this was a common feature of *Spn* capsular polysaccharide.

10.1128/mBio.02516-21.3 FIG S1 Representative transcytosis post-assay experiment; 0.5% Evans blue was added to the upper Transwell chamber and incubated for 1 h at 37°C. The OD_620nm_ of medium from the lower layer was measured and compared to a reference Evans blue standard. Download FIG S1, PDF file, 0.02 MB. Copyright © 2021 Brissac et al. 2021 Brissac et al. https://creativecommons.org/licenses/by/4.0/ This content is distributed under the terms of the Creative Commons Attribution 4.0 International license.

### Capsule increased intracellular survival by increasing tolerance to oxidative stress.

**FIG 2:**
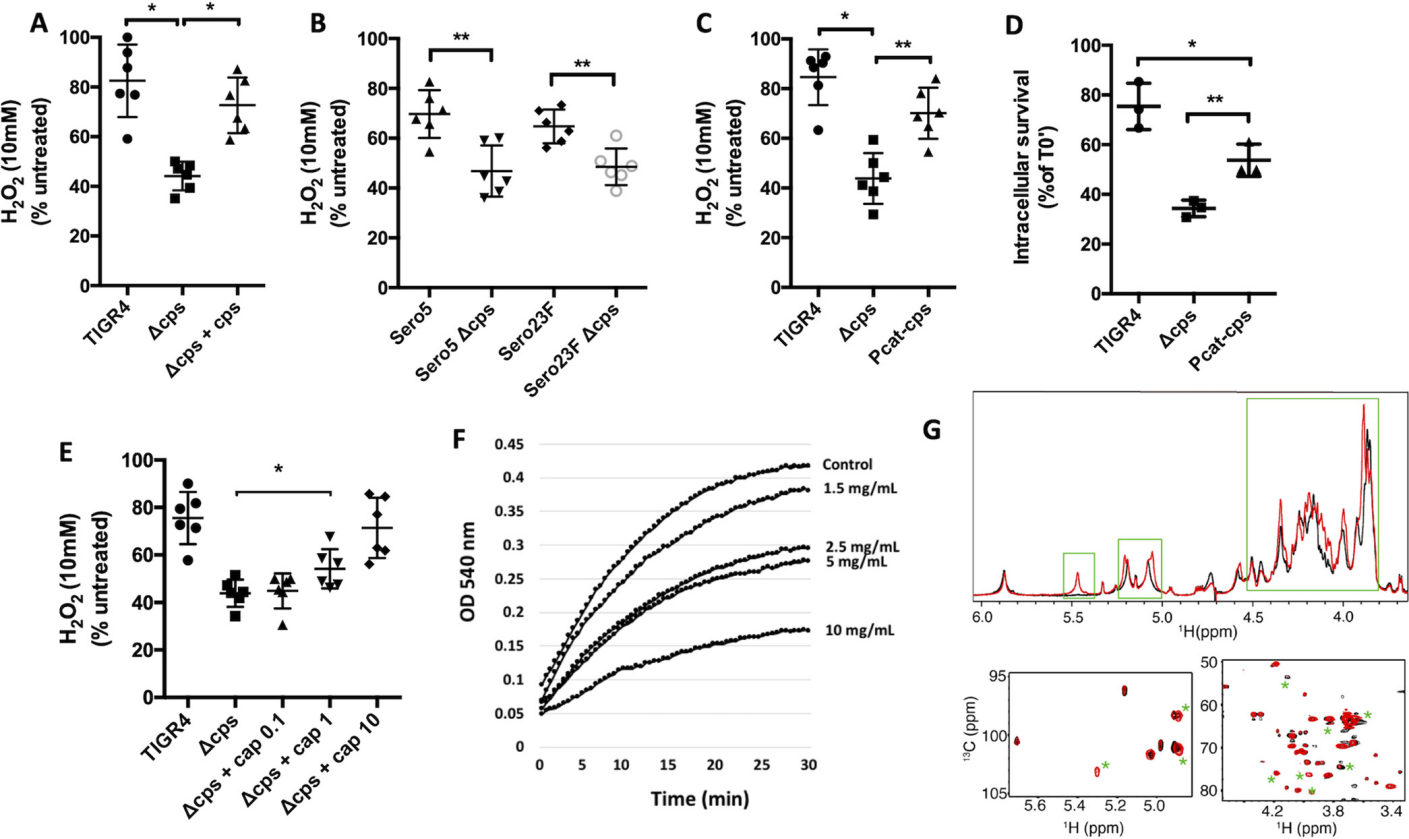
Capsule confers resistance to oxidative stress killing. (A and B) Capsule production reduces H_2_O_2_-mediated killing of (A) TIGR4 and (B) serotype 5 and 23F clinical isolates. Resistance to H_2_O_2_-mediated killing was determined by calculating the number of live *Spn* in THY supplemented with 10 mM H_2_O_2_ compared to *Spn* incubated in THY alone after 15 min of incubation. (C and D) Levels of capsule production impacted (C) *Spn* resistance to H_2_O_2_ and (D) intracellular survival within MCEC. Pneumococci with the promoter element *Pcat* upstream of the capsule operon (Pcat-*cps*) produced half the capsule of WT TIGR4 (see Fig. S3). (E) Exogenous polysaccharide at 1 mg/ml or higher protected unencapsulated TIGR4 (Δcps) from H_2_O_2_-mediated killing *in vitro*. (F) Purified capsule showed antioxidant properties in an NBT reduction assay. (G) ^1^H NMR (upper) and 2D 1H-13C HMQC spectra (lower) for untreated (black) and H_2_O_2_-treated (red) samples of serotype 4 polysaccharide at 50°C. Peaks marked with green boxes or asterisks denote significant spectral changes. Each dot is a biological replicate. Errors bars represent the standard error of the mean. Statistical analyses: Mann-Whitney U-test (panels A to E); one-way ANOVA with Tukey’s multiple comparison (panel D). Asterisks denote statistical significance: *, *P < *0.05; **, *P < *0.01.

Following clathrin-mediated endocytosis, bacteria within phagosomes are exposed to multiple stressors meant to kill and degrade cargo; this includes reactive oxygen species (ROS) (32, 33). Using bacterial culture medium supplemented with 10 mM H_2_O_2_, a dose previously used to test antimicrobial activity *in vitro* (34), we observed that the presence of capsule conferred up to a 30-min delay in *Spn* killing (Fig. 2A and B, Fig. S2). To investigate whether the amount of polysaccharide present on the pneumococcal surface affected resistance to ROS, we constructed a mutant harboring a constitutive promoter (Pcat) upstream of the capsule operon (Pcat-*cps*) that was comparably weaker than the native version found in WT TIGR4 (35). A fluorescein isothiocyanate (FITC)-dextran exclusion assay confirmed that Pcat-*cps* produced about half the amount of capsule produced by WT TIGR4 (Fig. S3). The reduced amount of capsule produced by Pcat-*cps* was enough to protect intracellular *Spn* compared to Δ*cps*, despite being more susceptible to killing than WT TIGR4 (Fig. 2C). Additionally, and *in vitro*, the protection conferred by the Pcat-*cps* mutant when treated with H_2_O_2_ was significantly less than WT TIGR4 (Fig. 2D). We subsequently hypothesized that encapsulated bacteria were less susceptible to ROS through a buffering mechanism whereby oxygen-derived free radicals preferentially attacked the polysaccharide strands. In support of this hypothesis, we observed that addition of purified serotype 4 capsule protected Δ*cps* from H_2_O_2_ *in vitro* in a dose-dependent manner (Fig. 2E) and, moreover, confirmed that the capsule was acting as a free radical scavenger using a nitroblue tetrazolium (NBT) reduction assay. In this instance, the addition of capsular polysaccharide also affected the kinetics of NBT reduction in a dose-dependent manner (Fig. 2F). We also performed one- and two-dimensional nuclear magnetic resonance (NMR) of purified type 4 capsular polysaccharide exposed to H_2_O_2_ for 3 h and 30 min (Fig. 2G, Fig. S4). Both experiments showed that exposure to H_2_O_2_ resulted in conformational changes as would be expected if capsule was acting as an antioxidant. These occurred at more than one site, suggesting it was more than one specific biochemical moiety that was responsible for this effect.

10.1128/mBio.02516-21.4 FIG S2 Resistance to H_2_O_2_-mediated killing over time was determined by calculating the number of live *Spn* in THY medium supplemented with 10 mM H_2_O_2_. Errors bars represent the standard error of the mean. Statistical analyses: two-way ANOVA with repeated measures. Asterisks denote the indicated statistical significance. Download FIG S2, PDF file, 0.02 MB. Copyright © 2021 Brissac et al. 2021 Brissac et al. https://creativecommons.org/licenses/by/4.0/ This content is distributed under the terms of the Creative Commons Attribution 4.0 International license.

10.1128/mBio.02516-21.5 FIG S3 FITC-dextran exclusion confirmed production of a reduced capsule amount of TIGR4 expressing *cps* locus under the control of Pcat promotor (Pcat-*cps*). Each point represents an individual bacterium. Statistical analyses: Mann-Whitney U-test. Asterisks denote statistical significance: ****, *P < *0.0001. Download FIG S3, PDF file, 0.03 MB. Copyright © 2021 Brissac et al. 2021 Brissac et al. https://creativecommons.org/licenses/by/4.0/ This content is distributed under the terms of the Creative Commons Attribution 4.0 International license.

10.1128/mBio.02516-21.6 FIG S4 NMR analysis of type 4 capsule treated with 10 mM H_2_O_2_ for 30 minutes versus control. Reproducible differences (*n* = 2) in the analyses are boxed. Download FIG S4, PDF file, 0.09 MB. Copyright © 2021 Brissac et al. 2021 Brissac et al. https://creativecommons.org/licenses/by/4.0/ This content is distributed under the terms of the Creative Commons Attribution 4.0 International license.

We attempted to demonstrate that ROS neutralization within the *Spn*-laden endosome conferred protection to unencapsulated pneumococci. These efforts included treatment of MCEC with Tempol, a membrane-permeable superoxide dismutase mimetic, and coating of *Spn* and the outer host cell membrane, which becomes the inner membrane of the vesicle following *Spn* phagocytosis, with either polyethylene glycol-conjugated catalase or polyethylene glycol-conjugated superoxide dismutase. However, we saw no impact of the treatments on rates of VEC translocation (Tempol versus control, *n* = 4, *P = *0.5351; conjugated catalase versus control, *n* = 6, *P = *0.1928; conjugated superoxide dismutase versus control, *n* = 6, *P = *0.3314). As ROS is an established antimicrobial factor within endosomes of cells (32, 33), one explanation for these negative results is that capsule also confers protection against other noxious agents found within the maturing endosome that were not neutralized by our treatments. One such possibility is the antimicrobial peptide LL-37 (20).

### Serotype influences intracellular survival and escape.

**FIG 3:**
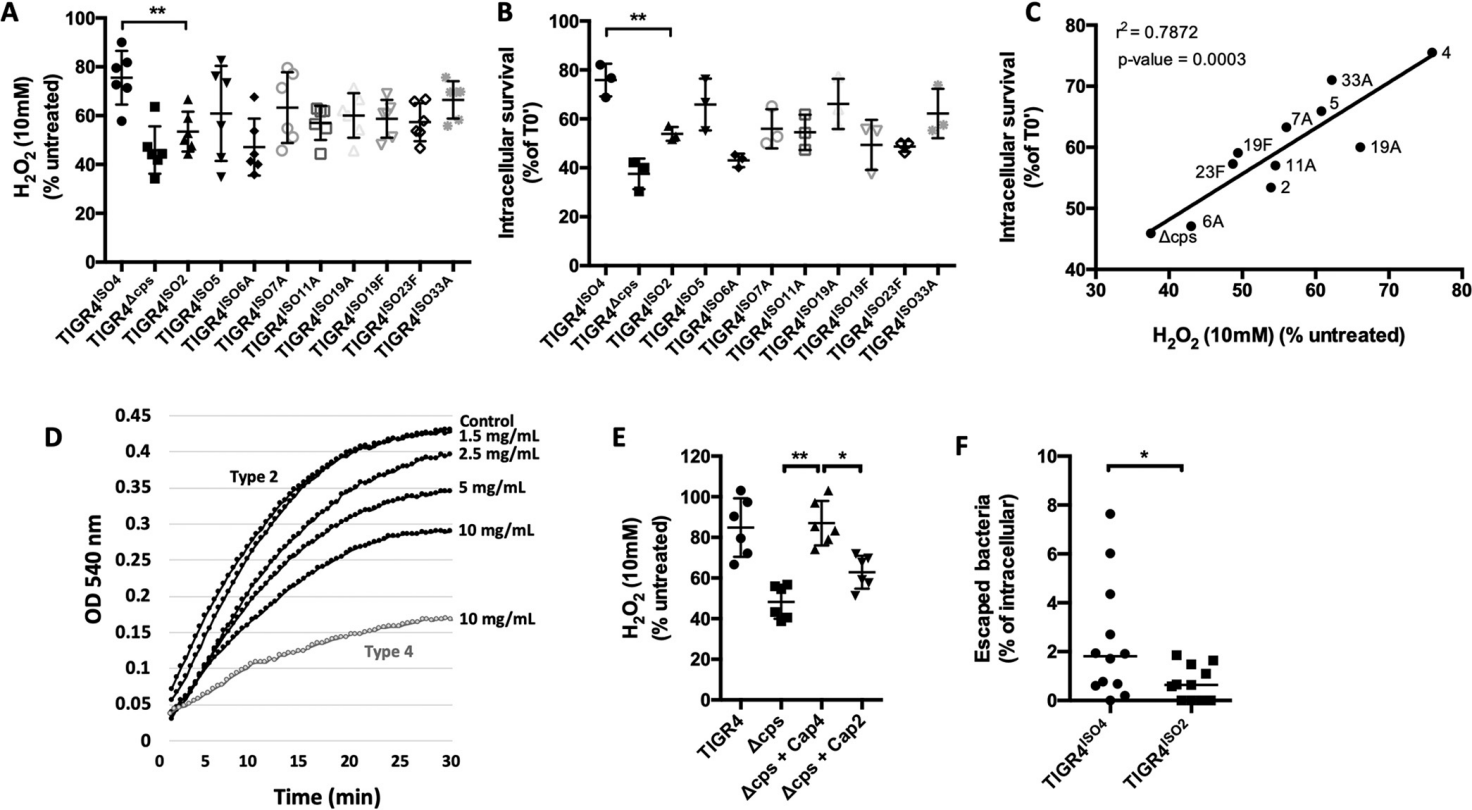
Capsule serotype influences resistance to oxidative stress killing. (A) Designated isogenic capsule switch mutants in the TIGR4 genetic background showed variable survival following incubation in THY supplemented with 10 mM H_2_O_2_. (B) The same 11-member panel showed considerable differences in intracellular survival within MCEC. (C) A positive correlation between resistance to H_2_O_2_ killing and intracellular survival was observed for these strains. (D) Serotype 2 capsule showed less antioxidant capability than serotype 4 capsule in an NBT reduction assay. Serotype 4 capsule at 10 mg/ml (gray line) was plotted as a reference. (E) Addition of exogenous type 2 was unable to protect Δ*cps* from H_2_O_2_ killing *in vitro*. (F) Moreover, TIGR4 expressing serotype 2 (TIGR4^ISO2^) also demonstrated a substantially reduced MCEC escape rate versus TIGR4 expressing its own serotype 4 capsule (TIGR4^ISO4^). Each dot is a biological replicate. Errors bars represent the standard error of the mean. Statistical analyses: one-way ANOVA with Tukey’s multiple comparison (panels A, B, D, and F), Spearman correlation (panel C), Mann-Whitney U-test (panel E). Asterisks denote statistical significance: *, *P < *0.05; **, *P < *0.01.

Clinical isolates of *Spn* belonging to different serotypes vary in the frequency in which they cause invasive disease (6, 7). To determine the importance of serotype on VEC translocation without the confounding effects of disparate genomes, we created a panel of isogenic capsule switch mutants of 10 clinically relevant serotypes in the genetic background of TIGR4, i.e., TIGR^ISO(serotype)^. As expected, we observed considerable variability in the capability of these capsule switch mutants to survive in medium supplemented with H_2_O_2_ (Fig. 3A) and survive within MCEC cells (Fig. 3B). Yet we also observed a very strong positive correlation between resistance to H_2_O_2_-mediated killing and intracellular survival of the tested isogenic mutants (Fig. 3C). Notably, when we compared the antioxidant properties of serotype 2, the serotype associated with midlevel resistance of TIGR4^ISO2^ to H_2_O_2_ and moderate intracellular survival rates, we found that capsule type 2 displayed consistently lower antioxidant activity than capsule type 4 (Fig. 3D). What is more, TIGR4^ISO2^ had half the MCEC escape frequency of TIGR4^ISO4^ (Fig. 3E). Thus, serotypes differed in the observed antioxidant property, and this positively correlated with rates of successful translocation. It is also noteworthy that resistance of the isogenic mutants to H_2_O_2_ killing in medium and intracellular survival also correlated positively with the known human attack rates for the represented serotypes (Fig. S5 and S6).

10.1128/mBio.02516-21.7 FIG S5 Resistance to H_2_O_2_-mediated killing of isogenic capsule switch mutants correlates with the attack rate of the corresponding serotypes. An association was tested for using Spearman’s rank correlation test. Each dot represents the average escape rate for the tested isolate after three biological replicates. Download FIG S5, PDF file, 0.03 MB. Copyright © 2021 Brissac et al. 2021 Brissac et al. https://creativecommons.org/licenses/by/4.0/ This content is distributed under the terms of the Creative Commons Attribution 4.0 International license.

10.1128/mBio.02516-21.8 FIG S6 Intracellular survival rates of the capsule switch mutants correlate with the published attack rate of the corresponding serotype. An association was tested for using Spearman’s rank correlation test. Each dot represents the average escape rate for the tested isolate after three biological replicates. Download FIG S6, PDF file, 0.03 MB. Copyright © 2021 Brissac et al. 2021 Brissac et al. https://creativecommons.org/licenses/by/4.0/ This content is distributed under the terms of the Creative Commons Attribution 4.0 International license.

### Capsule impacts disease presentation.

**FIG 4:**
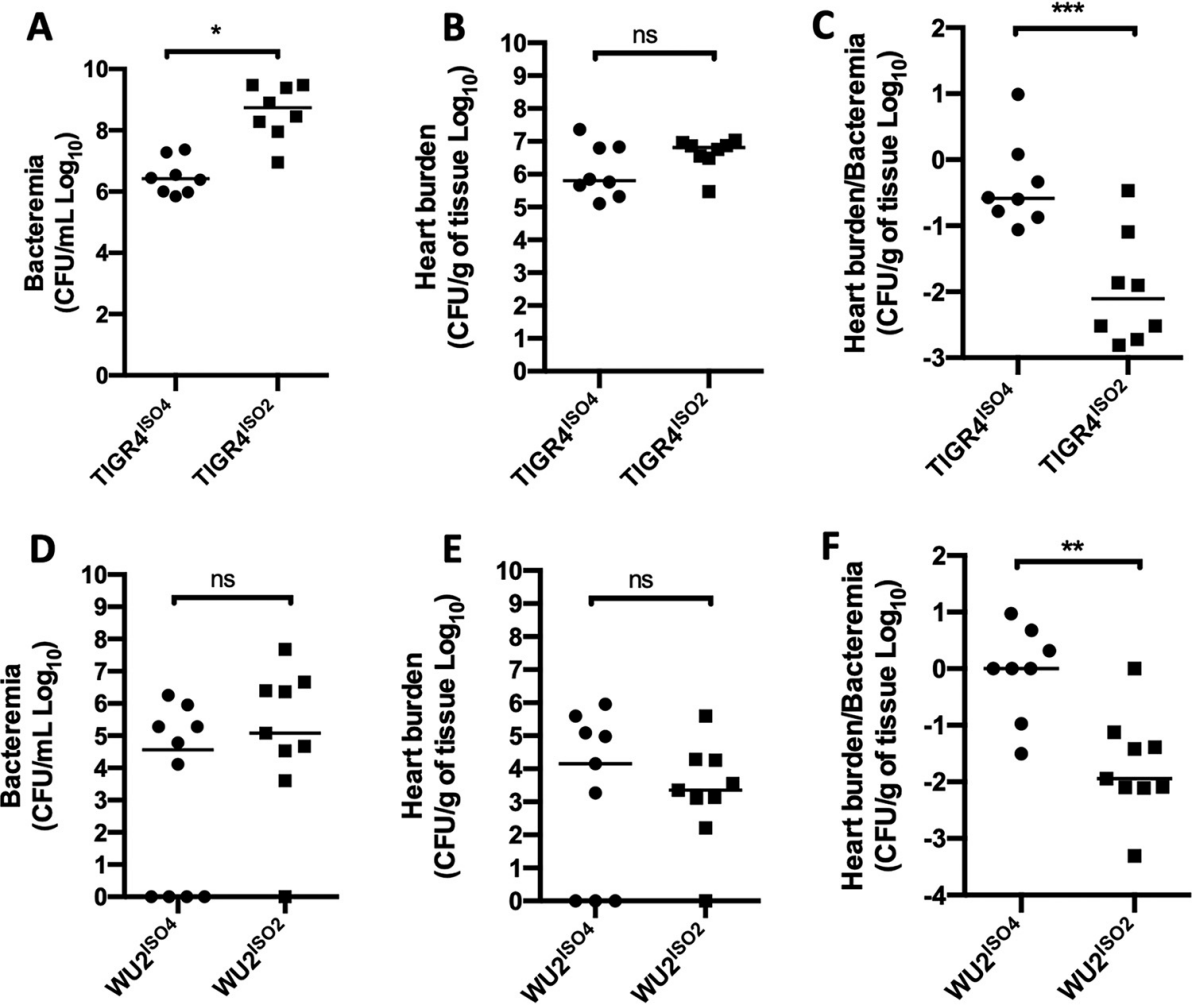
Capsule impacts disease presentation. (A, B, D, and E) C57B6 mice were challenged intraperitoneally with 10^5^ CFU of the designated isogenic capsule switches in the TIGR4 and WU2 genetic backgrounds, and (A and D) bacteremia and (B and E) heart titers were determined at the time of sacrifice (*T*_0_ + 36 h∼42 h). (C and F) The ability of these strains to escape from the vasculature was determined by normalization of heart titer by the level of bacteremia in paired samples. Each dot is a biological replicate. Errors bars represent the standard error of the mean. Statistical analyses: Student’s *t* test. Asterisks denote statistical significance: ns, not significant; *, *P < *0.05; **, *P < *0.01; ***, *P < *0.001.

As VEC translocation is a key step in *Spn* organ invasion during bacteremia, our results thus far suggest capsule-type would have a direct effect on this process *in vivo*. To test this possibility, we intraperitoneally (i.p.) challenged mice with TIGR4^ISO4^ or TIGR4^ISO2^ and examined the ability of each strain to translocate into the myocardium. This challenge model was chosen, as bacteria in the peritoneum continuously enter the circulation via the lymphatic system, avoiding the bottleneck that occurs in the spleen following intravenous injection (36), and myocardial invasion requires VEC translocation from the bloodstream (27). Following challenge, mice infected with TIGR4^ISO4^ had bloodstream titers 100-fold lower than those challenged with TIGR4^ISO2^ (Fig. 4A), yet equivalent numbers of TIGR4^ISO4^ and TIGR4^ISO2^ were recoverable from perfused hearts of these mice (Fig. 4B). When normalized against the number of bacteria in the blood, TIGR4^ISO4^ was ∼50-fold more efficient at invading the heart than TIGR4^ISO2^ (Fig. 4C). To rule out the possible positive effects of the matched genetic background and serotype for TIGR4^ISO4^, we also generated and tested isogenic mutants in a serotype 3 genetic background. WU2 expressing capsule types 4 and 2, WU2^ISO4^ and WU2^ISO2^, respectively, showed results similar to those in the TIGR4 genetic background (Fig. 4D to F). Notably, there were no significant differences in bacterial titers of mice infected with the WU2^ISO2^ versus WU2^ISO4^, which was not the case for TIGR4^ISO2^ and TIGR4^ISO4^, a finding that suggests factors carried by TIGR4 may interact with capsule in ways that alternatively promote or impair bloodstream survival.

### Capsule promotes transcytosis of other invasive pathogens.

**FIG 5:**
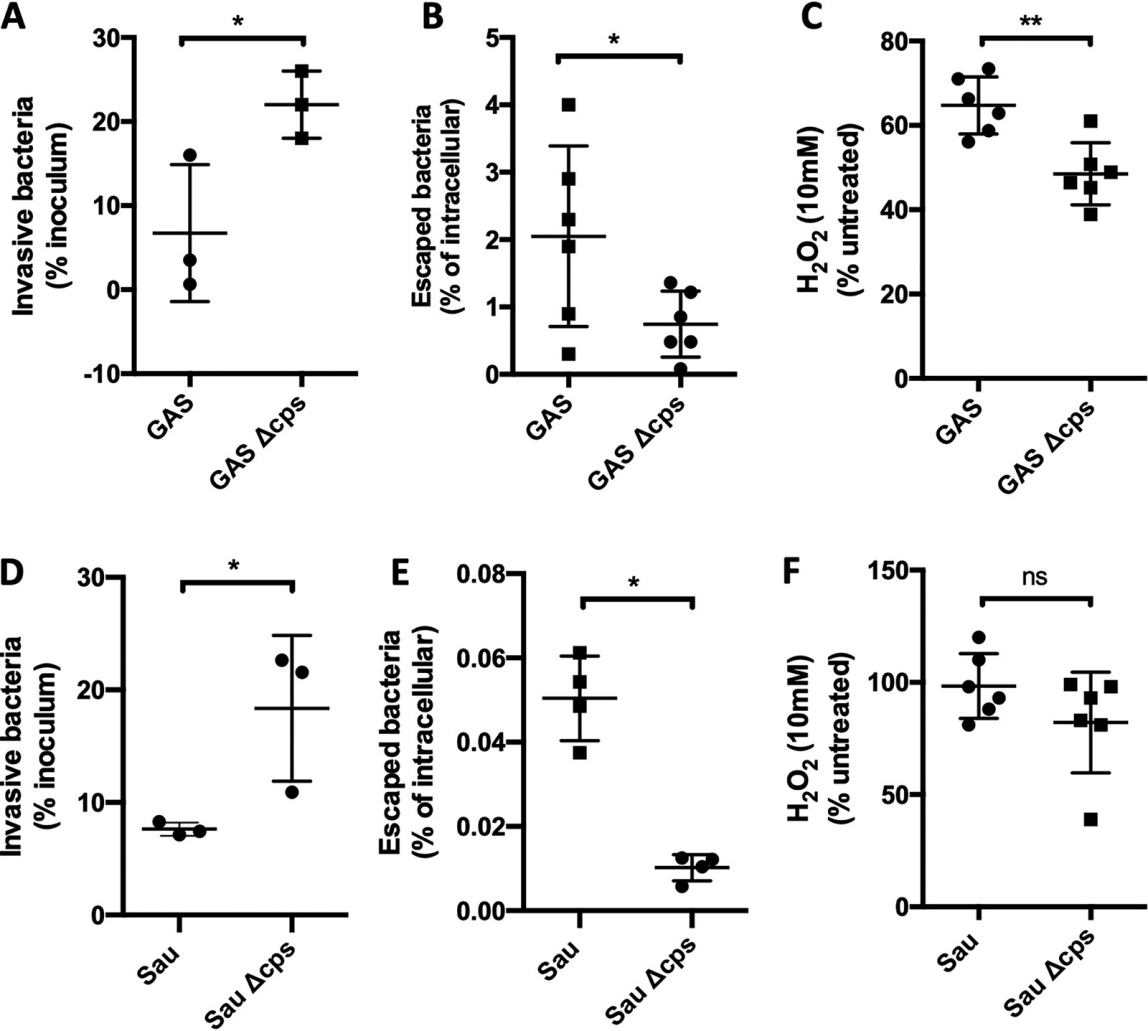
Capsule promotes transcytosis of other encapsulated pathogens. (A and B) Despite being outnumbered within the cells following (A) an invasion assay, (B) encapsulated S. pyogenes (GAS) escaped from within MCEC at higher rates than a nonencapsulated isogenic mutant (GAS Δ*cps*). (D and E) Similar results were observed for S. aureus. (C) GAS resistance to H_2_O_2_-mediated killing *in vitro* was capsule dependent, whereas for (F) S. aureus, deletion of capsule had no effect. Each dot is a biological replicate. Errors bars represent the standard error of the mean. Statistical analyses: Mann-Whitney U-test. Asterisks denote statistical significance: ns, not significant; *, *P < *0.05; **, *P < *0.01.

The production of a polysaccharide capsule is a common feature among many different bacteria capable of causing invasive disease. To test if our observations extended to other pathogens, we explored the role of the capsule on intracellular protection and translocation of Streptococcus pyogenes and Staphylococcus aureus. Both pathogens escaped from MCEC at higher rates than their respective nonencapsulated isogenic mutants, despite being initially outnumbered within the cells (Fig. 5A and B). For S. pyogenes we observed that the presence of capsule conferred resistance to H_2_O_2_-mediated killing, whereas this was not the case for S. aureus. The latter result is most likely due to the production of catalase by S. aureus. These results, along with those from *Spn*, indicate that capsule not only has an important role in protecting pathogenic bacteria against reactive oxygen species within the VEC endosome, but also protects against other noxious agents.

## DISCUSSION

Despite progress made with new vaccines and increasing access to intensive critical care, invasive bacterial diseases remain a leading cause of human morbidity and death worldwide (37, 38). Bacteremia, which can result in sepsis, also results in bacterial invasion of tissues (39). The latter alone can result in serious organ-specific complications. Examples include liver abscesses (40, 41), kidney damage and failure (42), cardiac microlesions and adverse cardiac events (27, 43, 44), and meningitis (45, 46). Thus, it is imperative to improve our understanding of the molecular mechanisms underlying organ invasion in order to identify prophylactic strategies and prevent associated damage.

Studied for over a century, capsule is one of *Spn*’s primary virulence determinants. The principal roles for capsule are to resist bacterial entrapment in mucus during colonization and prevent killing by host immune cells (11, 47). So far, 100 distinct serotypes of *Spn* capsule have been identified (5), with serotype-specific resistance to complement deposition demonstrated to be one reason why certain serotypes have a higher propensity to cause IPD (11). Other factors related to capsule that impact the propensity of *Spn* to cause disease are its abundance and negative charge, which both impair its association with host cells (48). Importantly, and although required for survival within the bloodstream (22), other than the demonstration that capsule is generally inhibitory of bacterial adhesion and invasion, how capsule influences *Spn* translocation across VEC following internalization was up to this point unknown.

Given capsule’s inhibitory effect on bacterial adhesion and therefore uptake by host cells, studies of *Spn* interactions with VEC have relied heavily on the use of nonencapsulated mutants (16, 18, 49–51). We suspect this is the principal reason why the role of capsule as an intracellular antioxidant has not been previously described. A few reports did use encapsulated bacteria to study pneumococci translocation across VEC, concluding that there was a meaningful impact. Fuchs et al. found that encapsulated *Spn* crossed human brain microvascular endothelial cell monolayers more efficiently than its unencapsulated counterparts (52). However, this study focused solely on serotype 7F and did not examine the mechanism or include findings with animal models. Ring et al. also described that different serotypes crossed an *in vitro* blood-brain barrier model with various efficiencies (25). However, Ring’s experiments emphasized the relevance of the genetic background, showing that phase variation, known to modulate capsule and other surface determinants (12, 19), was a major factor in *Spn*’s ability to cause meningitis. Here, to overcome the confounding effect of genetic variability between strains with different capsule types, we used isogenic capsule switch mutants to study the contribution of capsule alone in the fate of *Spn* once within the cell. Notably, Zaragoza et al. showed that polysaccharide enlargement by the fungus Cryptococcus neoformans conferred resistance to oxidative stress *in vitro* and concluded that this was a potential mechanism for intracellular survival within environmental predators (53). Relevant parallels between our findings and those of Zaragoza et al. include that capsule conferred its protective effect in dose-dependent fashion and that H_2_O_2_ altered capsule structure. These collective findings, as well as our observation of enhanced intracellular killing for unencapsulated S. aureus and S. pyogenes within VEC, suggest this trait of polysaccharide capsule is taken advantage of by a wide range of pathogenic microbes. Importantly, other biochemical studies of polysaccharides indicate they are indeed efficient free radical scavengers (54, 55).

Our experiments revealed that the capsule has a vital role during transcytosis through VEC layers by, if not preventing, at least delaying *Spn* intracellular killing. Within VEC, *Spn* encounters a hostile environment with the presence of microbicidal factors such as free radicals, antimicrobial peptides, lytic enzymes, and low pH. These agents/conditions act simultaneously, and we postulate that this is why we could not protect unencapsulated *Spn* within VEC from killing by blocking ROS generation or its neutralization with antioxidants. It is most likely for the same reason that unencapsulated S. aureus was less efficient at MCEC escape, despite the fact it did not require capsule for resistance to H_2_O_2_-mediated killing *in vitro*. Consistent with this notion, other investigators have shown that capsule protects *Spn* against the cationic peptide LL-37 (20), and LL-37 is present within phagolysosomes (56).

Not all serotypes of *Spn* tested were equally capable of conferring protection against H_2_O_2_ *in vitro*. This result was not unexpected given the considerable biochemical diversity of different serotypes (13). Along such lines, type 4 capsule was observed to confer superior antioxidant capabilities and resistance to intracellular killing versus type 2. The biological importance of this feature in pneumococcal pathogenesis was subsequently evidenced by results in mice that showed enhanced cardiac invasion by type 4-carrying pneumococci versus type 2 in both a TIGR4 (serotype 4) and a WU2 (serotype 3) genetic background. Further support for the importance of serotype effects on VEC translocation was the fact that resistance to H_2_O_2_ killing seen in the panel of isogenic mutants positively correlated with the published attack rates in humans for the tested serotypes.

The exact manner by which capsule conferred resistance to oxidative stress remains an open question and most likely varies considerably between serotypes based upon their biochemical composition. This notion is supported by our observation of multiple conformational changes in H_2_O_2_-exposed capsule by NMR. Studies are warranted to identify which features of capsule confer protection not only against reactive oxygen species, but also against other host defense factors found within the endolysosome. Nevertheless, we have gained important insights. Foremost, the amount of capsule produced matters. This was evidenced by our observation that reductions in capsule reduced the protective effects measured. We also now know that an exogenous source of capsule can protect unencapsulated *Spn* against ROS. While the antioxidant effects of exogenous capsule may not be critical during VEC translocation, it may have a vital role during colonization of the nasopharynx when *Spn* forms biofilms, and each pneumococcus carries less bacteria but is surrounded by an extracellular matrix that includes capsule (57, 58). We also know that capsules antioxidant effects are not absolute, and killing does occur over time. This suggests that capsule’s role during VEC translocation is to delay killing versus promoting long-term intracellular occupancy, the former providing pneumococci with more time to complete the translocation process. As protection is not absolute, we do not propose that the antioxidant effect of capsule is a means for the pneumococcus to avoid killing by macrophages or neutrophils, although this requires testing. Importantly, these antioxidant properties seem to be distinct from other key features of the capsule—such as resistance to complement deposition and opsonophagocytosis. The *in vivo* importance and divergence of the latter phenotype was evidenced by our results in mice that showed capsule serotype had a strong impact on bacterial burden in the bloodstream following challenge with isogenic capsule switch mutants. Thus, the biochemical properties of each serotype may favor or disfavor certain forms of disease due to how it influences interactions with host components and cells.

One important caveat to our findings is that we used a killing concentration of 10 mM H_2_O_2_. While this dose has been used by other investigators to study *Spn* susceptibility to H_2_O_2_ killing (34), it is well above the maximum amount, ∼100 μM, thought to be present within a maturing endolysosome. Notably, in pilot studies we did not observe meaningful *in vitro* killing of *Spn* at lower doses of H_2_O_2_, nor have others (34). Along similar lines, extra physiological levels of H_2_O_2_ *in vitro* were also required to kill C. neoformans *in vitro* (53). Accordingly, our results caution against the interpretation that physiologically relevant levels of ROS are alone sufficient for the eradication of bacteria. In turn, they reinforce the notion that reactive oxygen species generated by the host cell most likely act alongside, possibly in synergy with, other antimicrobial factors to kill pathogens within the endolysosome of VEC.

Knowledge of this new antioxidant role for capsule can be used to our advantage. For example, we can now screen and identify the nonvaccine serotypes which are most likely to be problematic in the future should serotype shift in response to the vaccine continue. Similar studies using isogenic capsule-switch mutants can and should be performed with other invasive pathogens, such as group B streptococci, whose primary virulence determinant is also capsule. Experiments testing for correlations between serotype-conferred resistance to oxidative stress and other key steps of the pathogenic process, such as the ability to establish long-term colonization in the airway or survive desiccation on fomites, are also now warranted.

In summary, we have identified a new and key role for pneumococcal capsule in promoting invasive disease, that is, serving as an intracellular antioxidant that facilitates bacterial translocation across VEC. Our findings suggest specific biochemical attributes of capsule, which vary in serotype-dependent fashion, impart different levels of intracellular protection, and this helps to explain the propensity of different serotypes for human invasive disease. Our observations with S. pyogenes and S. aureus suggest our results are extendable to other polysaccharide-encapsulated pathogens. Capsule’s antioxidant role may be important at many other sites, and this warrants future investigation.

## MATERIALS AND METHODS

### Bacterial strains and growth conditions.

The strains used in this study are described in Table S1. Isogenic mutant derivatives of *Spn* serotype 4 strain TIGR4, were created using splicing overlap extension PCR as described and using the primers listed in Table S2. Isogenic capsule switches were constructed as described in the supplemental material and methods. Bacteria were grown in Todd-Hewitt broth with 0.5% yeast extract (THY), or on blood agar plates (Remel), in a humidified atmosphere at 37°C with 5% CO_2_. When necessary, chloramphenicol (4.5 μg/ml), erythromycin (0.5 μg/ml), kanamycin (200 μg/ml), and streptomycin (300 μg/ml) were added to the medium. Appropriate and equivalent levels of capsule production by the isogenic capsule switch mutants were confirmed by (i) overt visualization of capsule production following overnight growth on plates, (ii) a positive quelling reaction using corresponding serotype-specific antisera, and (iii) testing of representative isogenic capsule switch mutants, i.e., corresponding to serotypes 2, 4, 11, 19A, and 19F, for levels of capsule production using the FITC-dextran exclusion assay.

10.1128/mBio.02516-21.1 TABLE S1 Strains used in this study. Download Table S1, PDF file, 0.09 MB. Copyright © 2021 Brissac et al. 2021 Brissac et al. https://creativecommons.org/licenses/by/4.0/ This content is distributed under the terms of the Creative Commons Attribution 4.0 International license.

10.1128/mBio.02516-21.2 TABLE S2 Primers used in this study. Download Table S2, PDF file, 0.07 MB. Copyright © 2021 Brissac et al. 2021 Brissac et al. https://creativecommons.org/licenses/by/4.0/ This content is distributed under the terms of the Creative Commons Attribution 4.0 International license.

### Mouse experiments.

All mice experiments were reviewed and approved by the Institutional Animal Care and Use Committee at The University of Alabama at Birmingham (UAB; protocol IACUC-20175). Animal care and experimental protocols adhered to public law 89-544 (Animal Welfare Act) and its amendments, Public Health Services guidelines, and the Guide for the Care and Use of Laboratory Animals (U.S. Department of Health and Human Services). Female 6-week-old C57B6 mice (Jackson) were challenged with 1.0 × 10^5^ pneumococci by intraperitoneal (i.p.) injection in 20 μl or 100 μl phosphate-buffered saline (PBS). Blood for assessment of bacterial burden was obtained by tail bleeds (2 to 5 μL) every 12 h. At the final time point (*T*_0_ + 36 h∼42 h) or when deemed moribund, 100 μl of blood was collected via retro-orbital bleed from anesthetized mice for bacteremia determination. Mice were subsequently euthanized by CO_2_ asphyxiation, and death was confirmed by pneumothorax. Mice were perfused by cardiac puncture using PBS, and then the collected organs were washed thoroughly with PBS and homogenized in 1 ml of PBS for bacterial burden determination.

### Cell culture.

Mouse cardiovascular endothelial cells (MCEC) were grown in Dulbecco’s modified Eagle’s medium (DMEM; Corning) supplemented with 10% heat-inactivated fetal bovine serum (FBS; Atlanta Biologicals) and 1× penicillin/streptomycin solution (Cellgro; Corning) in a humidified atmosphere at 37°C and 5% CO_2_.

### Adhesion and invasion experiments.

MCEC cells were seeded at 5.0 × 10^5^ cells per well in 12-well plates. Experiments were performed as described using 1.0 × 10^7^ Spn (multiplicity of infection [MOI], 10) (31). Intracellular survival rates were determined by normalizing the number of intracellular bacteria at any given time point normalized by the number of intracellular bacteria after 1 h of incubation with a bactericidal concentration of gentamicin.

### Transcytosis assays.

First, 5.0 × 10^5^ MCEC cells were seeded on Transwell permeable inserts (12 mm, 3-μm pore-size; Costar) in 12 wells and incubated for at least 48 h at 37°C with 5% CO_2_. For each experiment, extra inserts were seeded to determine the number of intracellular bacteria. Then, 5.0 × 10^6^ *Spn* bacteria were added to the cells before centrifugation at 500 × *g* for 5 min and incubation for 30 min at 37°C with 5% CO_2_. Inserts were then washed three times with prewarmed PBS and incubated for 30 more minutes in DMEM at 37°C with 5% CO_2_. Gentamicin was then added at a bactericidal concentration (200 μg/ml) in the upper chamber and at a bacteriostatic concentration (20 μg/ml) in the lower chamber before incubation at 37°C with 5% CO_2_ for 1 h. Filters were moved to new plates and incubated for 1 extra hour in DMEM containing bacteriostatic concentrations of gentamicin. Extra inserts seeded to determine the number of intracellular bacteria were washed three times in PBS after gentamicin incubation for 1 h. Cells were lysed by addition of cold water and 15 min of incubation at 4°C before plating. The escape rate was determined by the number of CFU recovered in the lower chamber normalized by the number of intracellular bacteria. For experiments where we sought to neutralize ROS production within the endosome of MCEC that had engulfed *Spn*, cells were treated with Tempol at 1 mM for 1 h prior to challenge (59); alternatively, the surfaces of *Spn* and MCEC were coated with polyethylene glycol-conjugated catalase or superoxide dismutase. The latter was done by treating MCEC and *Spn* for 1 h with 40 U/ml of the glycol-conjugated enzyme prior to MCEC challenge (60).

### Stress tolerance assays.

*Spn* from an overnight culture on a blood agar plate was used to inoculate THY, and this preculture was incubated until the optical density at 620 nm (OD_620nm_) reached 0.3 to 0.4. For ROS assays, 5.0 × 10^7^ bacteria were mixed with 20 mM H_2_O_2_ or menadione in THY (final concentration, 10 mM). Stress tolerance was determined by counting the number of viable bacteria at any given time point normalized by the number of bacteria incubated in plain THY.

### FITC-dextran exclusion assay.

To quantify the capsule thickness, we measured the exclusion area of FITC-dextran (FD2000S; Sigma) (61). Briefly, *Spn* was cultured in THY medium until the OD_600nm_ reached 0.3 and was centrifuged at 3,000 × *g* for 10 min, and pellet was resuspended in 500 μl of PBS or 4% paraformaldehyde solution. Then, 18 μl of resuspension was mixed with 2 μl of FITC-dextran solution (10 mg/ml, final 1 mg/ml concentration). The mixed solution was put on a microscope slide and visualized with a Leica LMD6 microscope equipped with a DFC3000G monochrome camera at ×40 magnification. The obtained images were analyzed using ImageJ processing software.

### NBT reduction assay.

To test the antioxidant capability of *Spn* capsule, we used the NBT reduction assay coupled to NADH and phenazine methosulfate (PMS) (62). Briefly, the reaction was performed in 96-well plates using 200 μl per assay. A mix of NADH (166 μM) and NBT (43 μM) freshly prepared in phosphate buffer (40 mM, pH 7.6) was incubated for 2 min at room temperature, and NBT reduction was started by the addition of 2.7 μM PMS. The plates were read in an iMark microplate reader (Bio-Rad) at 37°C. The optical density was monitored at 550 nm every 30 s for 30 min. The antioxidant efficacy of the purified capsule was estimated by the capability of protection of NBT from reduction compared to the controls with no polysaccharide.

### NMR spectroscopy.

A control sample of type 4 capsule polysaccharide (ATCC, Manassas, VA) was prepared by dissolving ∼6 mg in 3 ml of Milli-Q water and dialyzed (dialysis tubing, 8,000 molecular weight cutoff) overnight in 4 liters of Milli-Q water. The sample was then lyophilized and dissolved in 0.5 ml of 99.99% D_2_O (Cambridge Isotope Laboratories). For polysaccharide oxidation, ∼6 mg of type 4 capsule polysaccharide was treated with 10 mM H_2_O_2_ (Alfa Aesar, Massachusetts) in a total 3 ml volume. The solution was incubated at 37°C for either 30 min or 3 h. The solution was then dialyzed overnight against 4 liters of Milli-Q H_2_O followed by lyophilization. The sample was then dissolved in 0.5 ml of 99.99% D_2_O. ^1^H-^1^H and ^1^H-^13^C NMR data were collected at 50°C on a Bruker Avance II (700 MHz ^1^H) spectrometer equipped with cryogenic triple-resonance probes, processed with NMRPipe (63), and analyzed with NMRView (64). Deuterated water signal was used as a reference.

### Statistical analyses.

All analyses, excluding the NMR, were performed in GraphPad Prism 8 (San Diego, CA). Data were plotted as the mean of three technical replicates unless otherwise noted. Biological triplicates were graphed with error bars denoting the mean and standard error of the mean. Comparisons between two groups within one independent variable were assessed by the unpaired Student’s *t* test when data were normally distributed or by the Mann-Whitney U test when data distribution was not normal. Comparisons between three or more groups were assessed by analysis of variance (ANOVA) with Tukey’s posttest unless otherwise noted in the figure legend. The dependency between two variables were assessed by Spearman correlation.
