# Supplementary figures and images for: Capsule Promotes Intracellular Survival and Vascular Endothelial Cell Translocation during Invasive Pneumococcal Disease
Source: mBio. 2021 Oct 12;12(5):e02516-21. doi: 10.1128/mBio.02516-21 (PMC8510516; doi:10.1128/mBio.02516-21)

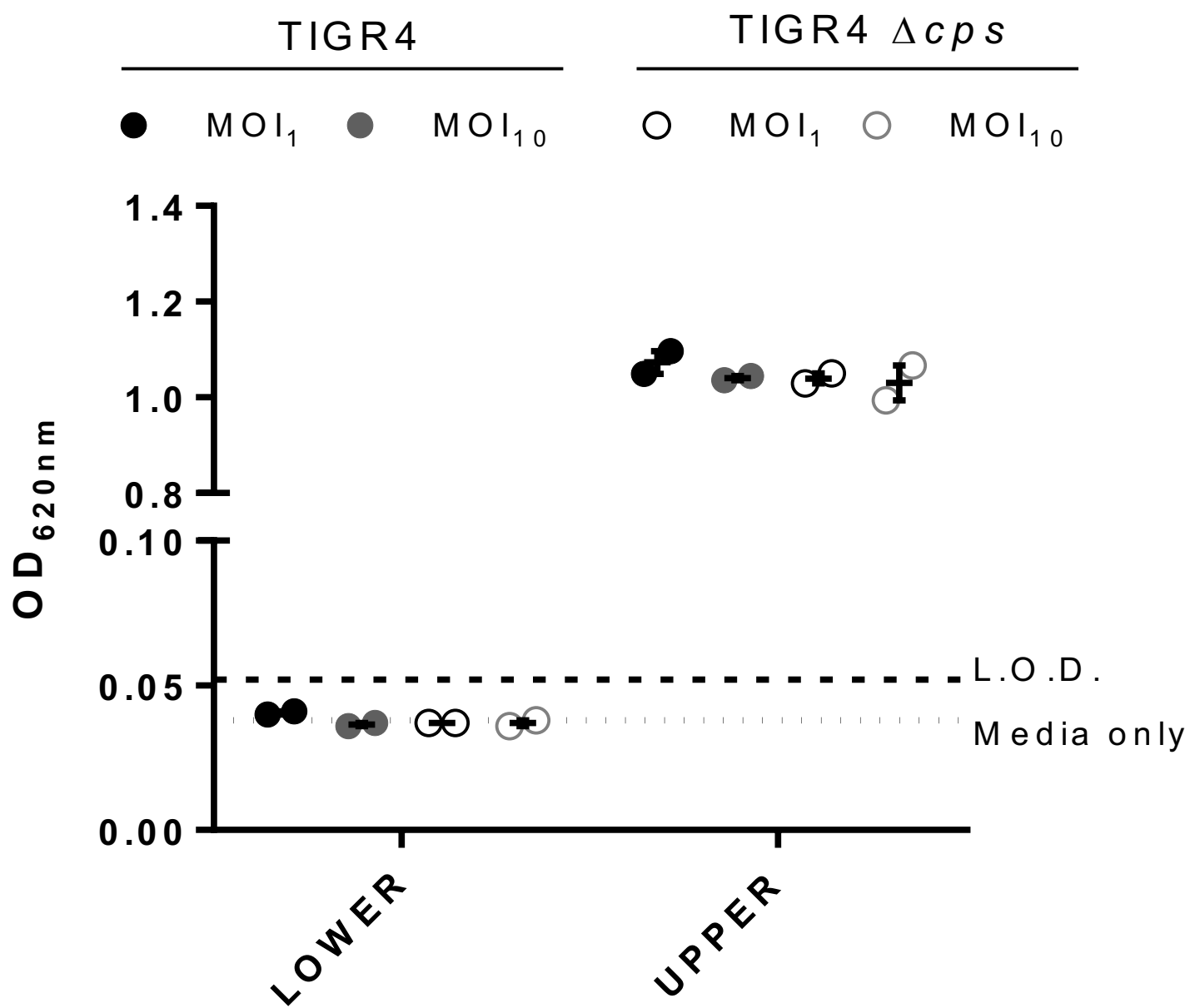

Supplement: FIG S1 [file mbio.02516-21-sf001.pdf]

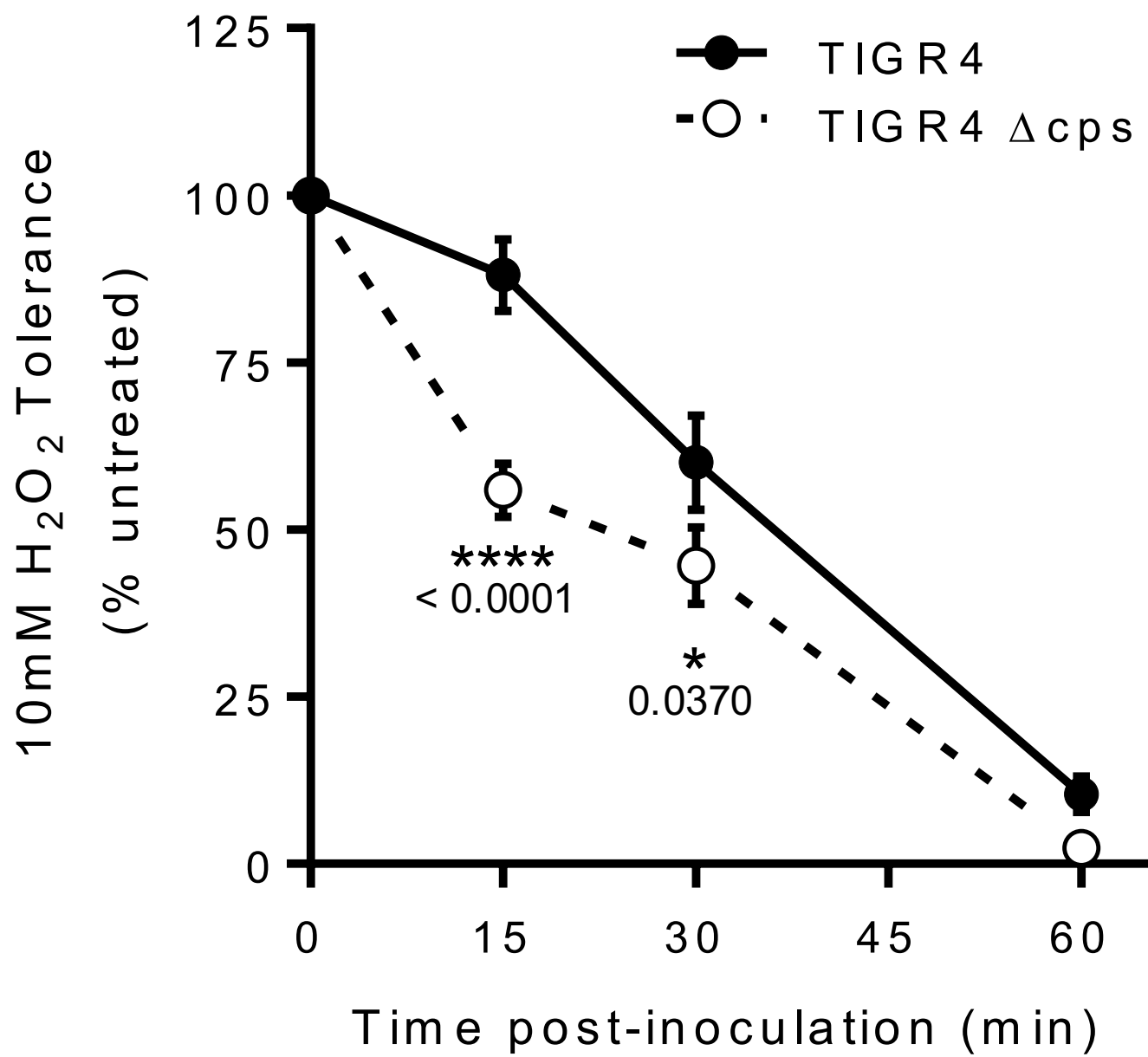

Supplement: FIG S2 [file mbio.02516-21-sf002.pdf]

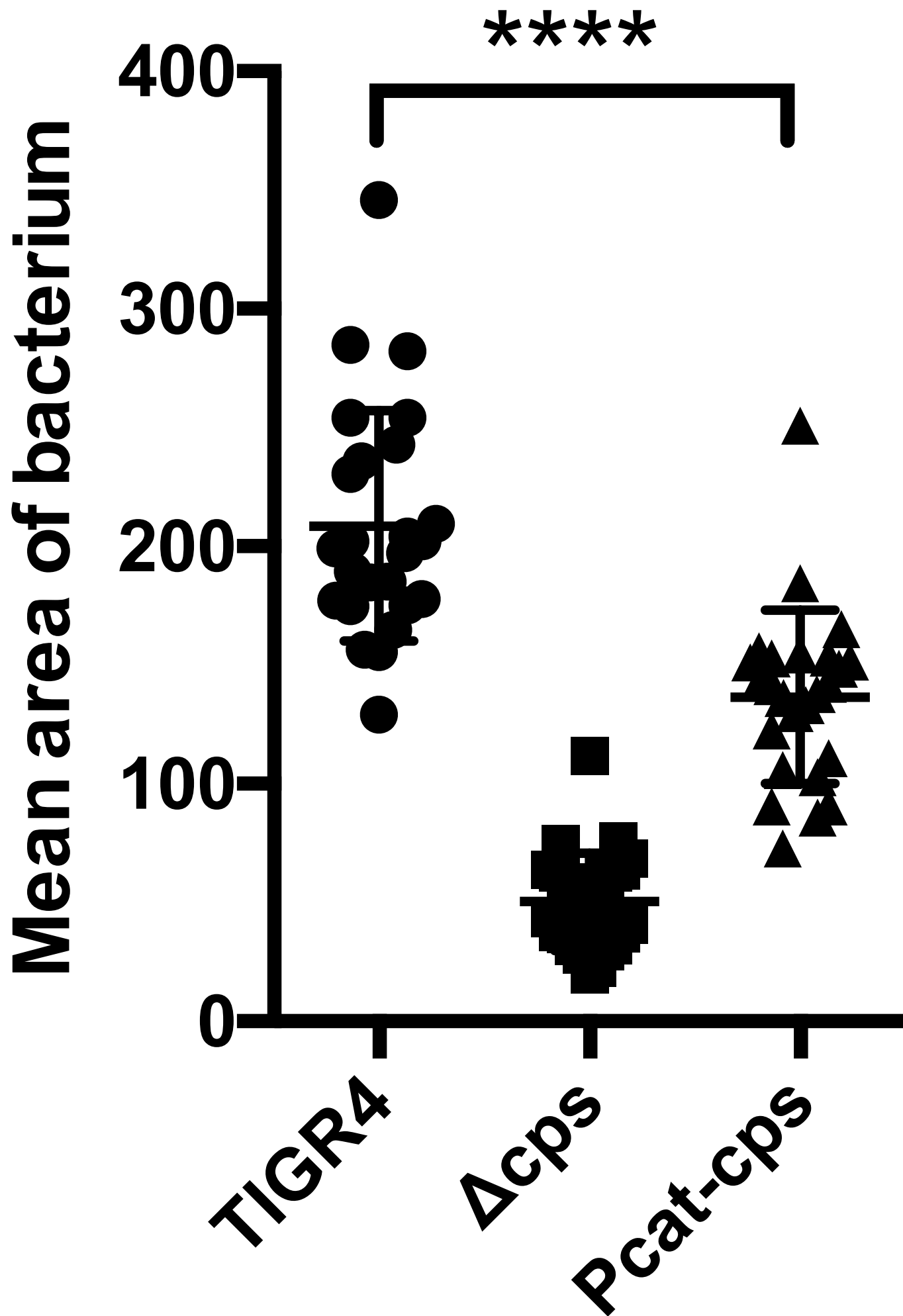

Supplement: FIG S3 [file mbio.02516-21-sf003.pdf]

Control (untreated)  
Control (treated)

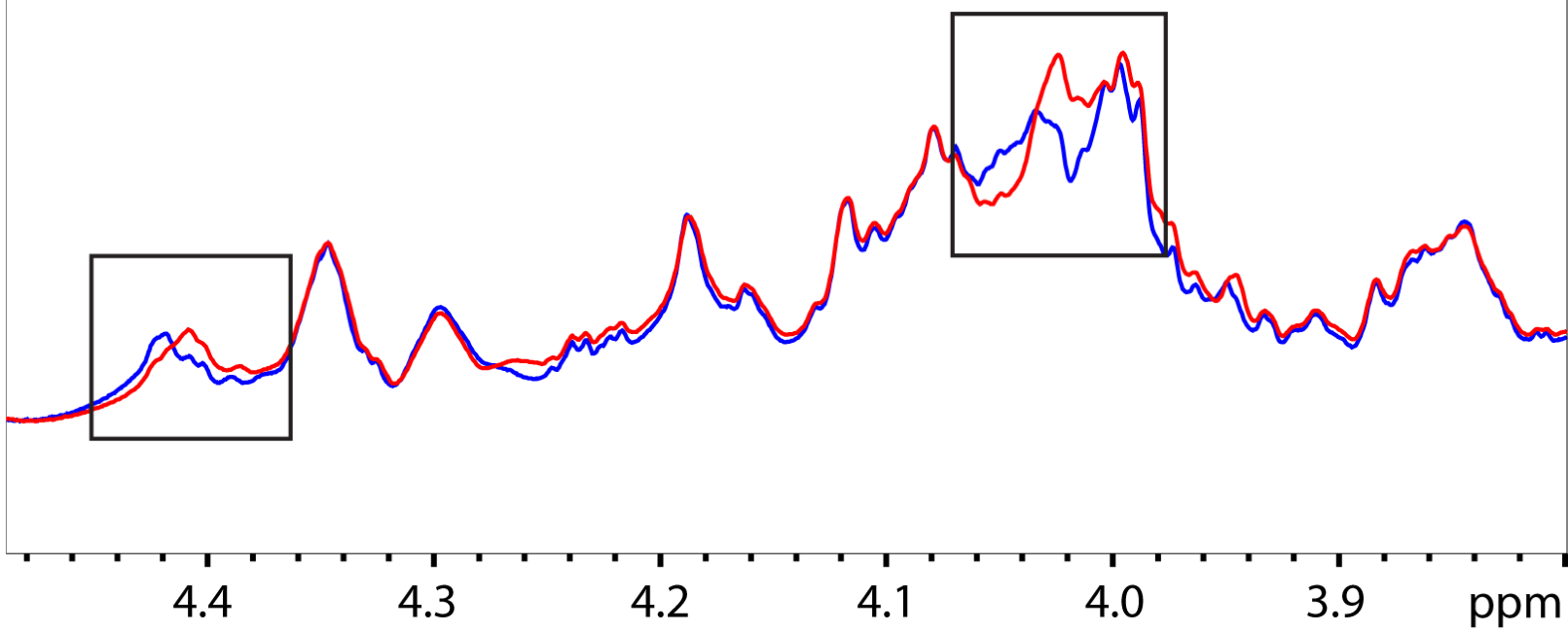

Supplement: FIG S4 [file mbio.02516-21-sf004.pdf]

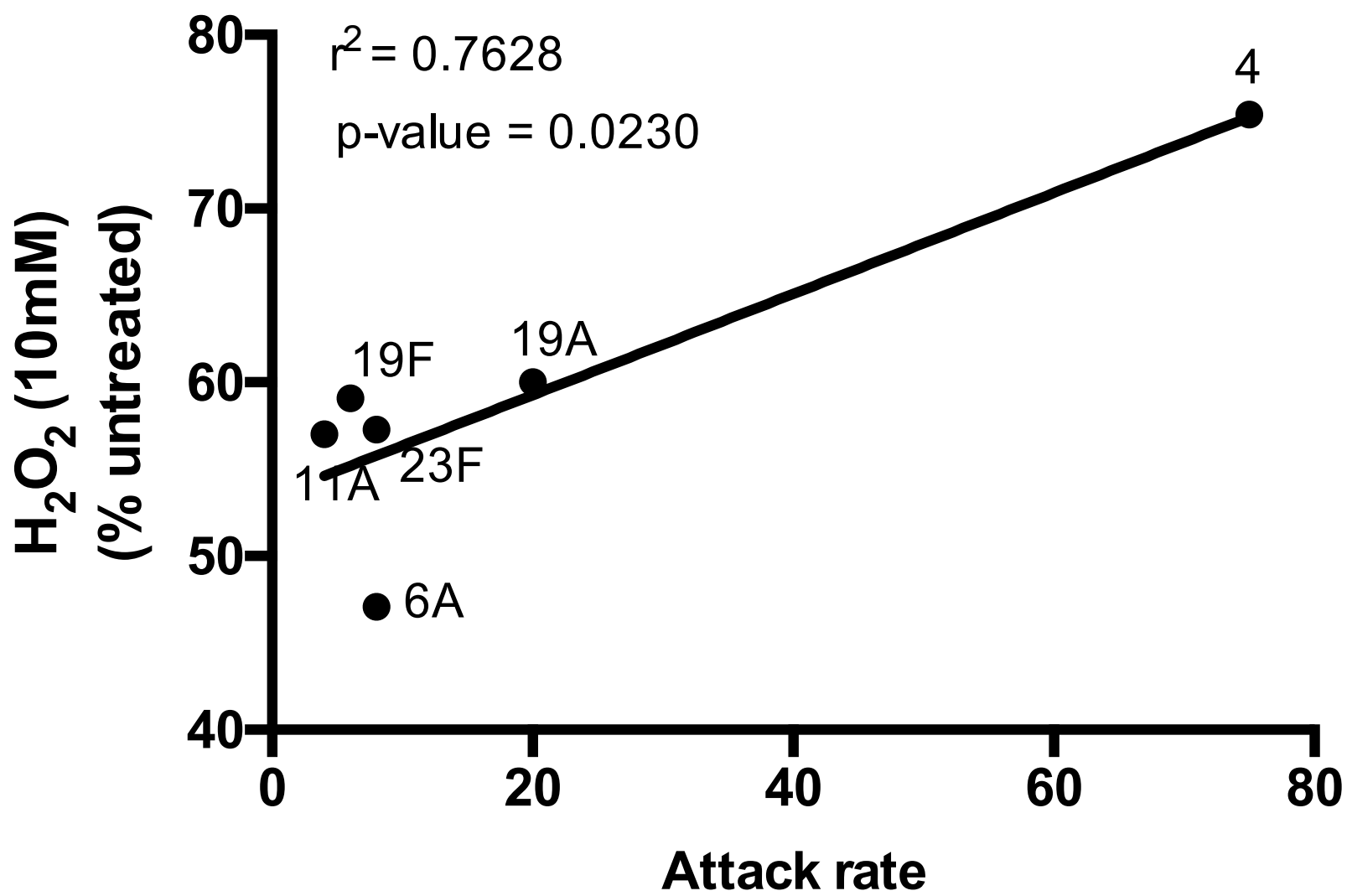

Supplement: FIG S5 [file mbio.02516-21-sf005.pdf]

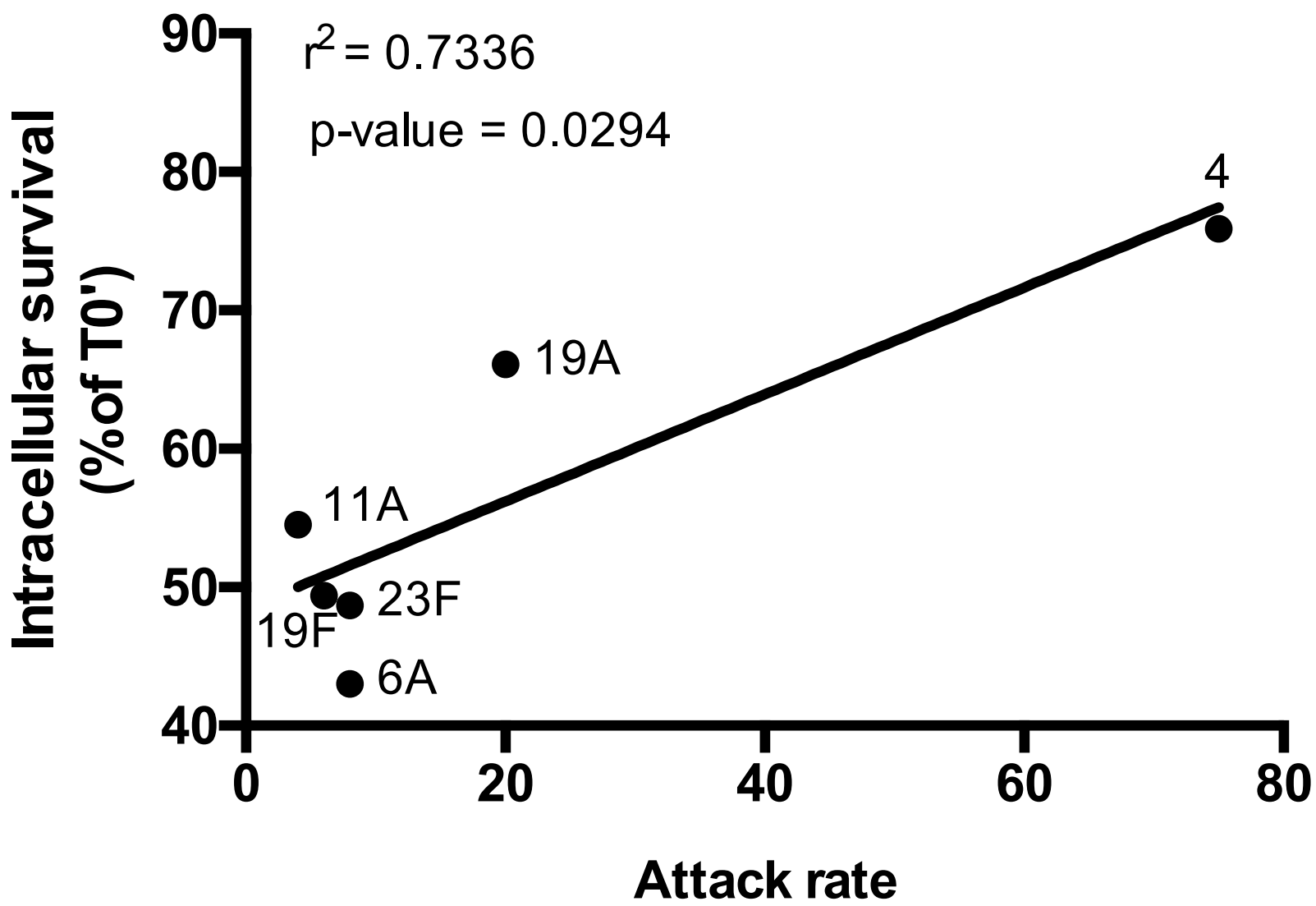

Supplement: FIG S6 [file mbio.02516-21-sf006.pdf]
