# Supplementary material for: Capsule Promotes Intracellular Survival and Vascular Endothelial Cell Translocation during Invasive Pneumococcal Disease
Source: mBio. 2021 Oct 12;12(5):e02516-21. doi: 10.1128/mBio.02516-21 (PMC8510516; doi:10.1128/mBio.02516-21)
Supplement: TABLE S1 [file mbio.02516-21-st001.pdf]

Supplemental Table 1. Strains used in this study

Strain Code	Strain Name	Relevant Genotype	Antibiotic resistance[a]	Relevant Features	Ref[b]
	TIGR4	WT	N/A	*Streptococcus pneumoniae serotype 4* Strain TIGR4	[1]
	WU2	WT	N/A	*Streptococcus pneumoniae serotype 3* Strain WU2	[2]
	D39	WT	N/A	*Streptococcus pneumoniae serotype 2* Strain D39	[3]
SpnTB4	Δ*cps*	TIGR4 *cps::cat*	Cm	Replacement of the whole cps locus by an Erythromycin resistance cassette	[4]
SpNTB25	Δ*lytA*	TIGR4 *ΔlytA::erm*	Ery	Replacement of the gene *lytA* by an Erythromycin resistance cassette	[4]
SpNTB102	Δ*lytA* Δ*cps*	TIGR4 *cps::cat ΔlytA::erm*	Cm Ery	Replacement of the gene *lytA* by an Erythromycin resistance cassette in a non-encapsulated background	[4]
SpNTB86	TIGR4^{ISO4}		Sm	TIGR4 expressing serotype 4 capsule	[4]
SpNTB88	TIGR4^{ISO2}		Sm	TIGR4 expressing serotype 2 capsule	[4]
SpNEM42	TIGR4^{ISO5}		Sm	TIGR4 expressing serotype 5 capsule	[4]
SpNTB45	TIGR4^{ISO6A}		Sm	TIGR4 expressing serotype 6A capsule	[4]
SpNTB59	TIGR4^{ISO7A}		Sm	TIGR4 expressing serotype 7A capsule	[4]
SpNEM12	TIGR4^{ISO11A}		Sm	TIGR4 expressing serotype 11A capsule	[4]
SpNEM13	TIGR4^{ISO19F}		Sm	TIGR4 expressing serotype 19F capsule	[4]
SpNEM14	TIGR4^{ISO19A}		Sm	TIGR4 expressing serotype 19A capsule	[4]
SpnTB62	TIGR4^{ISO23F}		Sm	TIGR4 expressing serotype 23F capsule	[4]
SpNTB97	WU2^{ISO4}		Sm	WU2 expressing serotype 4 capsule	[4]
SpNTB112	WU2^{ISO2}		Sm	WU2 expressing serotype 2 capsule	[4]
	BLS101		N/A	TIGR4 expressing 33A capsule	[5]
	BLS140	*ΔwciJ::aad9 wcJE::nptII-rpsL*	Kn Spc	TIGR4 expressing non O-Acetylated 33 capsule	[5]
	TIGRJS		Kn Sm	Non encapsulated TIGR4 containing janus cassette	[5]
1	MNK1178		N/A	Serotype 1 Clinical isolate	[4]
2	MNK0300		N/A	Serotype 5 Clinical isolate	[4]
3	MNK0009		N/A	Serotype 6B Clinical isolate	[4]
4	MNK0759		N/A	Serotype 7F Clinical isolate	[4]
6	MNK0418		N/A	Serotype 11A Clinical isolate	[4]
7	MNK0057		N/A	Serotype 15B Clinical isolate	[4]
10	MNK0361		N/A	Serotype 19F Clinical isolate	[4]
11	MNK0420		N/A	Serotype 23F Clinical isolate	[4]
	5448			Group A Streptococcus M1T1 clinical isolate 5448	[6]

5884ΔhasA	Ery	Capsule deficient mutant of 5448	[6]
Reynolds		*Staphylococcus aureus* Reynolds CP5 positive	[7]
JLO22		*Staphylococcus aureus* Reynolds CP5 negative	[7]

[a] Cm: Chloramphenicol (4.5µg/mL), Sm Streptomycin (300µg/ml), Kn: Kanamycin (200µg/mL), Ery: Erythromycin (0.5µg/ml, 5µg/ml for GAS)
[b] [1] (1), [2] (2), [3] (3), [4] this study, [5] (4), [6] (5) [7] (6)

1. C. K. Stover *et al.*, Complete genome sequence of Pseudomonas aeruginosa PAO1, an opportunistic pathogen. *Nature* **406**, 959-964 (2000).
2. D. E. Briles *et al.*, Antiphosphocholine antibodies found in normal mouse serum are protective against intravenous infection with type 3 streptococcus pneumoniae. *J Exp Med* **153**, 694-705 (1981).
3. J. A. Lanie *et al.*, Genome sequence of Avery's virulent serotype 2 strain D39 of Streptococcus pneumoniae and comparison with that of unencapsulated laboratory strain R6. *J Bacteriol* **189**, 38-51 (2007).
4. B. L. Spencer, J. S. Saad, A. T. Shenoy, C. J. Orihuela, M. H. Nahm, Position of O-Acetylation within the Capsular Repeat Unit Impacts the Biological Properties of Pneumococcal Serotypes 33A and 33F. *Infect Immun* **85** (2017).
5. A. Hollands *et al.*, Genetic switch to hypervirulence reduces colonization phenotypes of the globally disseminated group A streptococcus M1T1 clone. *J Infect Dis* **202**, 11-19 (2010).
6. M. Portoles, K. B. Kiser, N. Bhasin, K. H. Chan, J. C. Lee, Staphylococcus aureus Cap5O has UDP-ManNAc dehydrogenase activity and is essential for capsule expression. *Infect Immun* **69**, 917-923 (2001).
