# Supplementary material for: Capsule Promotes Intracellular Survival and Vascular Endothelial Cell Translocation during Invasive Pneumococcal Disease
Source: mBio. 2021 Oct 12;12(5):e02516-21. doi: 10.1128/mBio.02516-21 (PMC8510516; doi:10.1128/mBio.02516-21)
Supplement: TABLE S2 [file mbio.02516-21-st002.pdf]

Supplemental Table 2. Primers used in this study

Name	5' → 3' sequences	Description	Ref[a]
lytA_UP-FW<br>lytA_UP (Spl_EryK7)-RV<br>lytA_DN (Spl_EryK7)-FW<br>lytA_DN-RV	AGCCTCTTGTTGATTTATCG<br>TCTAAGTCTTATTCCCGGGCATATTCTACTCCTTATC<br>TCTAAATTTGGAAGCCGTTGATTACAGTAAAATAATAATG<br>TTTCTCTGATTTCCTCAACC	Construction of TIGR4 Δ*lytA*	[1]
CmK7-FW<br>CmK7-RV	GTATGTCAAAAGACCC<br>GCGGCCGCTTATAAAAGCC	Cassette amplification	[1]
EryK7-FW<br>Ery-K7-RV	CCCGGGAATAAGACTTAGA<br>CGGCTTCCAAATTTACA	Cassette amplification	[1]
cps4A_UP-FW<br>cps4A_UP (Spl_CmK7)-RV<br>Full_CAPS4_Locus_DN (Spl_CmK7)-FW<br>Full_CAPS4_Locus_DN-RV	ATCTAAACAGGTGCTAGG<br>GGTCTTTTGACATACCATGATTAATACCTATAC<br>GCTTTTATAAGCGGCCGCGTTTGGAGGAAAAGCTGAATTGTATA<br>TGGGACGGAGTAAAAGAGTCC	Construction of TIGR4 Δcps	[1]
licA_UP-FW<br>licA_UP (Spl_SpcK7)-RV<br>licA_DN (Spl_SpcK7)-FW<br>licA_DN-RV	TTGACTCTTGCTCATAGC<br>TCCTCCTCACTATTTGCACAATTAACCTCCAGT<br>TGGAAACACTTCGTGAATGGAGGTTCAGATGAAAAGTA<br>ATTAACCTGAACCAAGGC	Construction of TIGR4 Δ*licA*	[1]
rpsL-FW<br>rpsL-RV	GTG CTG ACA AAT GTT GC<br>TTC TCT TTA TCC CCT TTC C	Cassette amplification	[1]
Janus-FW<br>Janus-RV	GGG AGG ACT GTA TAA AA<br>GAA CCC GAT AAA ACT GA	Cassette amplification	[1]
dexB-FW<br>dexB -(Spl_Janus)-RV<br>aliA (Spl_Janus)-FW<br>aliA-RV	AAG TTA GGA AAC TAC GG<br>AGT CCT CCC GTT CAT CTT CTT TCT CC<br>ATC GGG TTC GGA ACA ATA GGT TGT GG<br>GAT TCA GCA TTC AAG GG	Construction of Spn_{JSK7}	[1]
2f<br>2r	TATCCCAGTTCAATATTTCTCCACTACACC<br>ACACAAAATATAGGCAGAGAGAGACTACT	Serotyping	[2]
3f<br>3r	ATGGTGTGATTTCTCCTAGATTGGAAAGTAG<br>CTTCTCCAATTGCTTACCAAGTGCAATAACG		[2]
4f<br>4r	CTGTTACTTGTTCTGGACTCTCGATAATTGG<br>GCCCACTCCTGTTAAAATCCTACCCGCATTG		[3]
Pcat-FW<br>Pcat-RV	ATTTGTTTGATTTTTAATGGATAATGTGATATAATGGTTCAATGTTCAATGTATAGGTATTAATCATGAGTAGACGT<br>TGAACCATTATATCACATTATCCATTAAAAATCAAACAAATTATTTTTACTATCTGCATCTTTAAGTATCTTAGTAGACTTCCC	Construction of Pcat-cps	[1]

[a] [1] This study, [2] (1), [3] (2)

1. M. da Gloria Carvalho *et al.*, Revisiting pneumococcal carriage by use of broth enrichment and PCR techniques for enhanced detection of carriage and serotypes. *J Clin Microbiol* **48**, 1611-1618 (2010).
2. R. Pai, R. E. Gertz, B. Beall, Sequential multiplex PCR approach for determining capsular serotypes of Streptococcus pneumoniae isolates. *J Clin Microbiol* **44**, 124-131 (2006).
